# Spatial and temporal movements in Pyrenean bearded vultures (*Gypaetus barbatus*): Integrating movement ecology into conservation practice

**DOI:** 10.1038/srep35746

**Published:** 2016-10-25

**Authors:** Antoni Margalida, Juan Manuel Pérez-García, Ivan Afonso, Rubén Moreno-Opo

**Affiliations:** 1 Department of Animal Science, Faculty of Life Sciences and Engineering, University of Lleida, 25198 Lleida, Spain; 2 Division of Conservation Biology, Institute of Ecology and Evolution, University of Bern, CH-3012, Bern, Switzerland; 3 Department of Applied Biology, University Miguel Hernández, E-03202 Elche, Spain; 4 Conselh Generau d’Aran, Passeg dera Libertat, 16 E-25530 Vielha, Spain; 5 Evolution and Conservation Biology Research Group, University Complutense of Madrid, E-28049 Madrid, Spain

## Abstract

Understanding the movement of threatened species is important if we are to optimize management and conservation actions. Here, we describe the age and sex specific spatial and temporal ranging patterns of 19 bearded vultures *Gypaetus barbatus* tracked with GPS technology. Our findings suggest that spatial asymmetries are a consequence of breeding status and age-classes. Territorial individuals exploited home ranges of about 50 km^2^, while non-territorial birds used areas of around 10 000 km^2^ (with no seasonal differences). Mean daily movements differed between territorial (23.8 km) and non-territorial birds (46.1 km), and differences were also found between sexes in non-territorial birds. Daily maximum distances travelled per day also differed between territorial (8.2 km) and non-territorial individuals (26.5 km). Territorial females moved greater distances (12 km) than males (6.6 km). Taking into account high-use core areas (K20), Supplementary Feeding Sites (SFS) do not seem to play an important role in the use of space by bearded vultures. For non-territorial and territorial individuals, 54% and 46% of their home ranges (K90), respectively, were outside protected areas. Our findings will help develop guidelines for establishing priority areas based on spatial use, and also optimize management and conservation actions for this threatened species.

Movement ecology is an emerging discipline that can play a critical role in the design and improvement of management and conservation measures that target threatened taxa. Thus, the link between movement ecology and biodiversity is a key factor in understanding ecological and evolutionary processes and in improving the efficacy of conservation efforts^1^,^2^. Several recent examples show the importance of movement ecology studies in topics such as age-related aspects of migration^3^, the energetics and timing of migration^4^, the conservation of migratory flyways^5^, the selection of stopover, wintering and breeding areas^6^,^7^,^8^,^9^, the influence of environmental conditions^10^, transboundary management policies^11^ and foraging behaviour^12^.

Age classes of most large territorial vertebrates segregate spatially as a consequence of their breeding status and the different ways in which they exploit food resources. For example, the foraging movements of pre-adult individuals during dispersal periods are typically aimed at finding food resources^13^ and so, due to this non-territorial lifestyle, patches with higher food availability are preferably selected by non-breeding individuals. This is a very important and sensitive period from a conservation perspective because pre-breeding dispersal in large vertebrates with deferred maturity usually lasts for several years, during which time individuals exploit large areas; their success in finding food will thus greatly affect their survival prospects^14^,^15^,^16^. Consequently, estimation of the use of space during these pre-settlement movements is a key element in the design of any adaptive management program designed to improve geographical expansion and restore viable metapopulations^17^,^18^.

By contrast, when individuals reach adulthood, settle and occupy breeding territories, their central-place foraging behaviour restricts their movements to the nest surroundings. Foraging movements of breeding individuals are linked to obtaining food resources but also include aspects related to habitat choice, functional responses and the trade-offs between food-seeking and safety^19^,^20^. Breeding status obliges individuals to pursue a cost-benefit equilibrium in their foraging strategy that takes into account the distance they need to cover and the benefits (i.e. food) they can obtain. This is more obvious for species such as avian scavengers that depend on spatially and temporally unpredictable food resources^21^,^22^. In these species, days may elapse before food is obtained and so individuals try to optimize their energetic gain at each feeding event at carcasses^23^,^24^.

The bearded vulture *Gypaetus barbatus* is a threatened species in the Western Palearctic, where it is restricted to mountain biomes such as the Pyrenees (*n* = 130 pairs, 61% of the European population in 2015) (A. Margalida, unpubl. data). Nevertheless, several ongoing reintroduction projects are currently being performed in certain subpopulations (i.e. Alps, Andalusia). The largest natural European population of bearded vultures is confined to the Pyrenees and knowledge of the movement of this species will help improve management actions aimed at increasing the distribution of this species and minimizing the risk of metapopulation extinction. The identification of the core areas this vulture uses is an important step in the conservation of the species and will improve management strategies. The difficulties inherent in the long-term monitoring of long-lived species greatly hinder the collection of reliable information on this subject. To date, information on the ranging behaviour of bearded vultures in the Pyrenees is limited to nine non-breeding individuals tracked using ARGOS satellite telemetry, which yielded 427 locations^25^, and a further nine non-breeding individuals monitored with GPS satellite telemetry^17^. Another study has been carried out on the subspecies *Gypaetus barbatus meridionalis* in South Africa, in which 18 individuals were monitored^26^. The current study aims to provide the first comprehensive understanding of the ranging behaviour of this vulture’s threatened Pyrenean population via the use of a larger sample size of all age-classes, tracked over a number of years/seasons with highly accurate GPS satellite technology.

Through an analysis of existing information on the pre-breeding dispersal and territorial movements of GPS-tracked bearded vultures in the Pyrenees, in this study we offer insights into the use of space by this endangered species. We analyzed the movements of 19 bearded vultures in the Pyrenees (Spain, France and Andorra) between 2006 and 2014 and were thus able to identify home range sizes and daily movements in terms of age, sex and period (breeding vs. non-breeding seasons). The nesting areas occupied by breeding individuals are commonly used as surrogates of critical areas for conservation and buffer areas are established around nesting sites on this basis^27^. However, from a conservation perspective, high-quality areas in which individuals obtain most resources should similarly be considered as vital in the design and implementation of conservation priorities. Accordingly, we predicted that, apart from inherent individual age-, sex- and breeding-status-related differences^28^, no substantial asymmetries would appear between the vultures’ life periods. Specifically, we predicted that movements in the non-breeding and breeding periods would be similar because the poor weather in winter is compensated for by a network of supplementary feeding sites (also known as vulture restaurants) that function between November and April^29^. On the other hand, the summer (non-breeding) period offers greater food availability during the transhumance season^30^. To date, the aim behind the management of the Pyrenean population of bearded vultures has primarily been to enhance the region’s environmental carrying capacity by installing a network of feeding sites, which have been found to play an important role in the observed movement patterns of the species^17^. Based on the data gathered, we here develop guidelines for the establishment of priority areas in which management and conservation actions targeting this threatened species should be concentrated.

## Results

We obtained a total of 66 467 GPS locations of 19 bearded vultures from 29 November 2006 to 31 December 2014 (Table 1). The mean monitoring period per bird was 1104.0 ± 789.3 days (range 29–2058), while the mean number of locations per bird was 3509.1 ± 2542.8 (range 57–7886). The distribution of locations was as follows: by sex 36.1% females and 63.9% males, and by age class 80.6% adults, 15.3% subadults, 3.3% immatures and 0.8% juveniles. During this period, six individuals died (two poisoned, two intoxicated by lead and two after collisions with power lines) and the transmitter was lost or failed in nine birds (Table 1).

### Daily movements

Non-territorial adults recorded the highest mean daily movements at 46.1 ± 41.4 km, the maximum distance travelled by a bearded vulture in one day being 259.5 km. Territorial adults moved a mean of 23.8 ± 20.9 km per day but with an average maximum distance reached from the initial point per day of 8.2 ± 12 km (see Table 2 for complete results; see also Supplementary Information Table S1). The maximum distance travelled by a tracked bearded vulture in a single day was 176.2 km from the initial point. Juveniles moved shorter distances, 8.4 ± 7.3 km, and their maximum distance travelled per day was 5.7 ± 5.2 km. Subadults and non-territorial adults moved similar average hourly distances of 4.5 ± 7.3 km per hour. Territorial adults moved an average hourly distance of 2.5 ± 3.9 km per hour; juveniles were the slowest age group with an hourly distance of 1.1 ± 2.1 km per hour (Table 2). We found significant differences between the mean daily distances travelled by territorial and by non-territorial birds (Wilcoxon test z = 10.4, p < 0.001) and likewise found differences between the maximum distances travelled per day by territorial and by non-territorial birds (z = 19.3, p < 0.001). Non–territorial birds moved to more distant areas than territorial birds (26.5 ± 27.5 km vs. 8.2 ± 12.0 km).

Differences between sexes were not detected in the mean daily distances travelled by territorial birds (z = 0.92, p = 0.35) but were found between sexes in non-territorial birds (z = −3.81 p < 0.001), as females travelled shorter distances (37.4 ± 34.0 km) than males (47.2 ± 44 km; Table 2). No sex-related differences in maximum distances travelled per day were found in non-territorial birds (z = −1.60, p = 0.11); territorial females moved further then than males (12.0 ± 18.2 km vs. 6.6 ± 7.6 km; z = 3.37, p < 0.001).

### Home range

**Figure 1:**
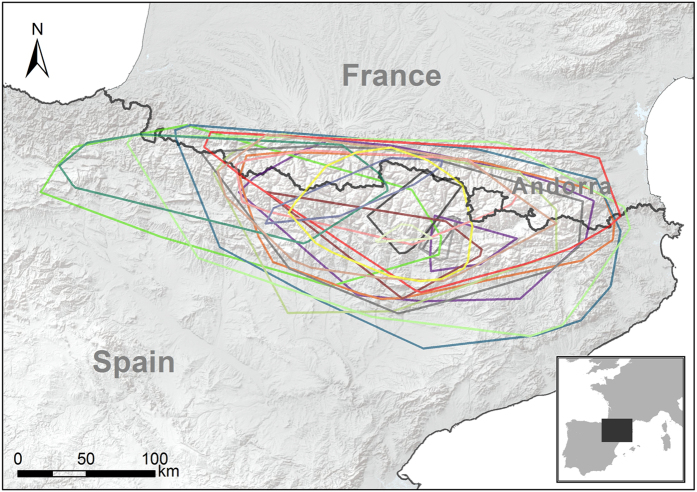
Minimum convex polygon used by 19 GPS-tracked bearded vultures *Gypaetus barbatus* in the Pyrenees from 2006 to 2014. Maps were created in Rstudio 0.99 (https://www.rstudio.com) and R 3.0.3 (http://www.R-project.org) in combination with ArcGIS 9.1 (http://www.esri.com/ see Material and Methods).

**Figure 2:**
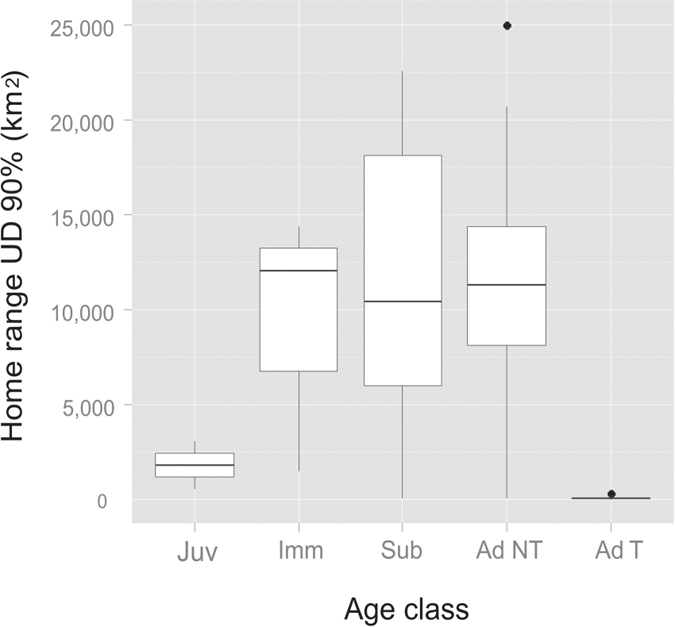
Home range size and shape by age class. We show UD kernel at 90% of juvenile (Juv), immature (Imm), subadult (Sub), non-territorial adult (Ad NT) and territorial adult (Ad T) of bearded vultures *Gypaetus barbatus* tracked in the Pyrenees from 2006 to 2014.

**Figure 3:**
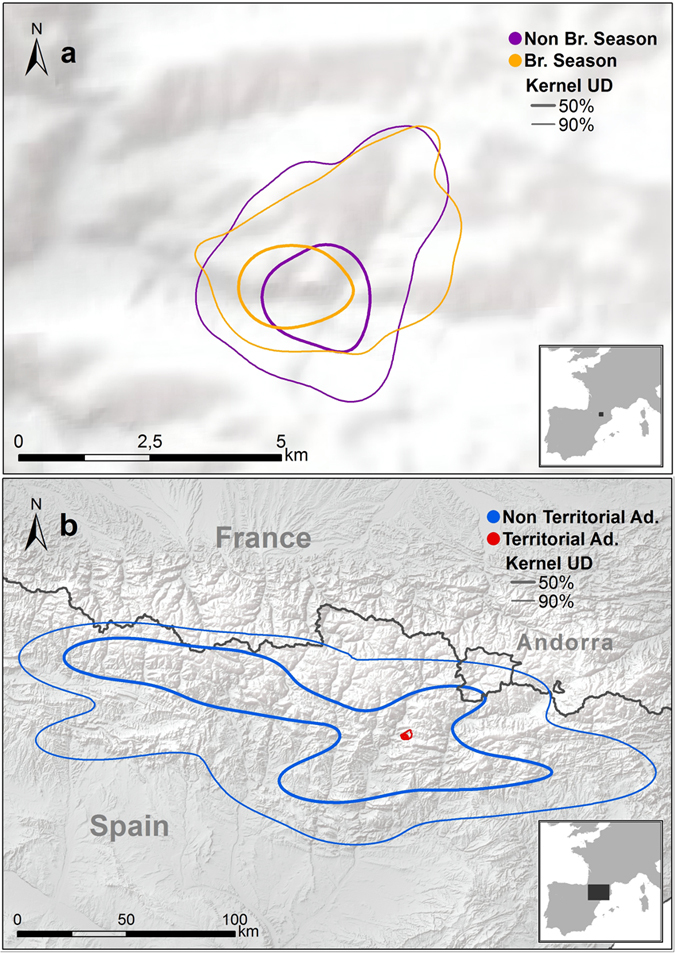
Bearded vulture *Gypaetus barbatus* ranging behaviour tracked in the Pyrenees from 2006 to 2014. We show utilization density areas UD kernel 90% (thick line) and UD kernel 50% (thin line) for (**a**) territorial adult in the breeding season (yellow) and non-breeding season (violet) and (**b**) we show yearly UD areas for a non-territorial adult (blue) versus a territorial adult (red). Maps were created in Rstudio 0.99 (https://www.rstudio.com) and R 3.0.3 (http://www.R-project.org) in combination with ArcGIS 9.1 (http://www.esri.com/ see Material and Methods).

The total area used by all tracked bearded vultures was 48 738.6 km^2^ (Minimum Convex Polygon, MCP; Fig. 1), which encompassed the whole of the current breeding range of the species in the Pyrenees. Annual bearded vulture home ranges showed important differences between age classes (see Table 3 for complete data, Figs 2 and 3, and Supplementary Information Table S2). These differences were statistically significant in the core area (Wilcoxon test z = 6.41, p < 0.001), home range (z = 6.36, p < 0.001) and MCP (z = 5.97, p < 0.001). Territorial individuals exploited home range areas of about 50 km^2^, while non-territorials birds used areas of around 10,000 km^2^. When assessing home range differences between non-territorial age classes, we only found significant differences between juveniles and non-territorial adults in all home range estimators (K50 z = −1.94, p = 0.05; K90 z = −2.04, p = 0.041; MCP z = −1.99, p = 0.045; Fig. 3). Differences between sexes in home range size were found in territorial adults but not in non-territorial birds (K50 z = −0.49, p = 0.63; K90 z = −1.14, p = 0.26; MCP z = −1.22, p = 0.22). Territorial males used smaller core areas than territorial females (z = 2.01, p = 0.043) (see Table 3). No sex-related differences were found in MCP or home range in territorial adults (K90 z = 1.57, p = 0.12; MCP z = 0.74, p = 0.45).

**Figure 4:**
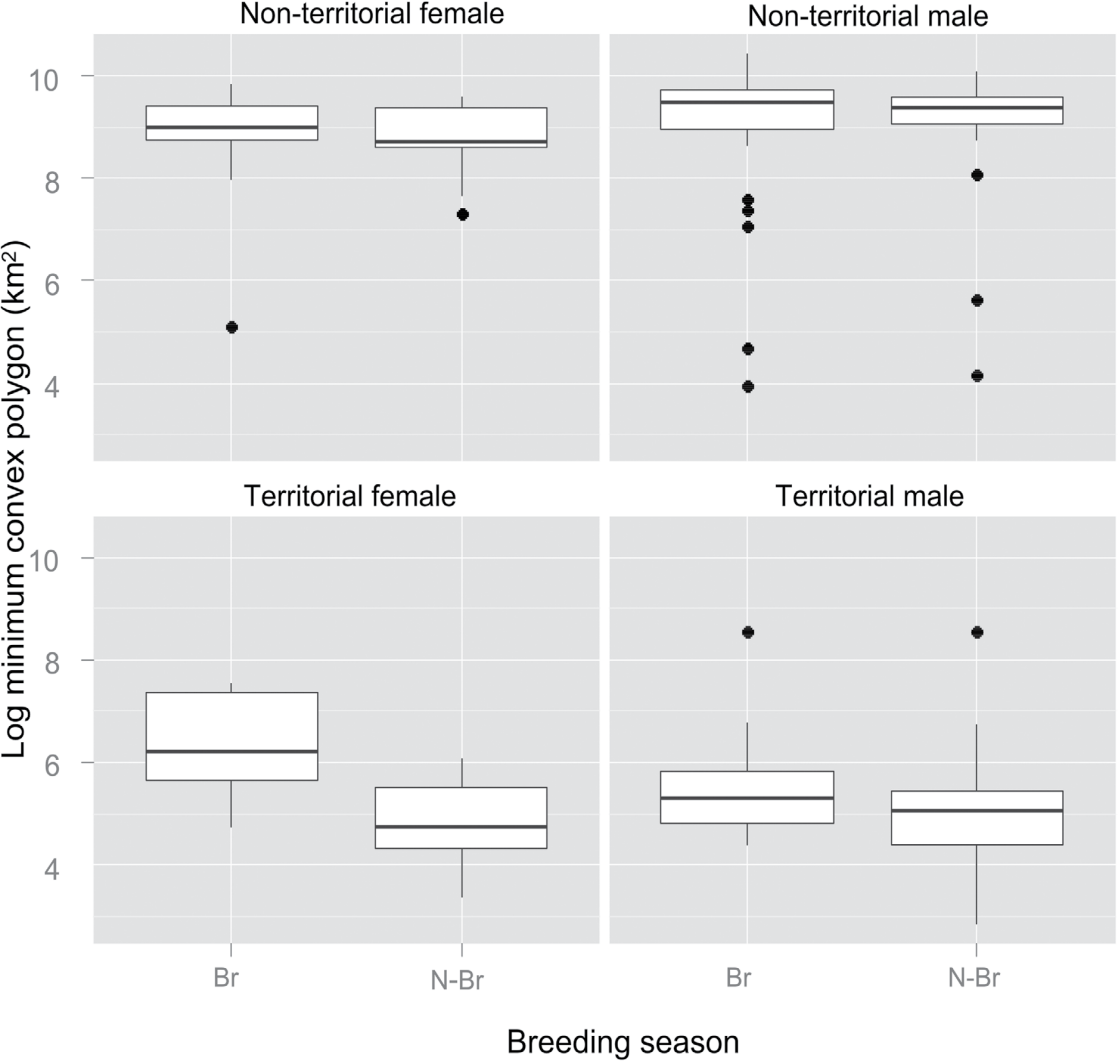
Minimum convex polygon (log transformed) of territorial adults vs non-territorial bearded vultures *Gypaetus barbatus* tracked in the Pyrenees from 2006 to 2014 comparing breeding (Br) vs. non-breeding season (N-Br) and sex (male and female).

No seasonal differences were detected in the home range size of non-territorial birds (K50 z = −0.50, p = 0.62; K90 z = −0.29, p = 0.77; MCP z = 0.50, p = 0.61) or territorial adults (K50 z = −0.45, p = 0.65; K90 z = −0.05, p = 0.95), with the exception of the MCP (MCP z = 1.96, p = 0.049; Fig. 4). Territorial adult females used larger MCP areas during the breeding season (z = 2.01, p = 0.044) compared to the non-breeding season. By contrast, no differences were found in home range (z = 0.18, p = 0.85) or core area (z = 0.18, p = 0.85). Territorial males showed no differences in any home range estimator (K50 z = −0.55, p = 0.58; K90 z = −0.04, p = 0.96; MCP, z = 1.14, p = 0.25).

### Factors related to home range size

At core-area scale, territorial status, age, interaction between sex, territorial status and the full model including all the variables performed better than the null model. At home-range scale, all of these factors were included, together with two other factors (season and the interaction between season and territorial status). For territorial birds at core-area scale, season was the only factor that improved the null model (see Supplementary Information Table S3). At home-range scale, no factors improved the null model. For non-territorial individuals, age was the principal factor related to the size of their home and core ranges. In relation to the pairwise comparison between age classes in the core area, juvenile birds showed significant differences to the other age classes (see Table 4).

### Overlapping foraging range with protected areas

In all, 43.8% of the home range of non-territorial birds (K90 22 723.6 km^2^) was within protected areas. A similar overlap percentage, 44.5%, was found for the core area (K50 6669.9 km^2^). Overlap with protected areas was higher in territorial birds, with 54.3% of the home range (K90 838.7 km^2^) and 65.7% of the core area (K50 158.9 km^2^) lying within protected areas.

## Discussion

This study provides the first description and comparison of spatial and temporal ranging patterns of territorial and non-territorial Pyrenean bearded vultures tracked with GPS technology (see also refs 17 and 25). Such high-quality information will help optimize management and conservation actions for this threatened species in the context of the European metapopulation. It may also assist in assessing food availability in the area used by the species, as well the functionality of the SFS designed to enhance survival, breeding success and geographical expansion^31^,^32^,^33^. Thus, actions aimed at 1) increasing the range of the species; 2) establishing SFS; and 3) minimizing the detrimental effects of territory shrinkage should be based on available data on range movements since almost no geographical expansion of this population has occurred in the last few decades^31^.

The bearded vulture, an obligate avian scavenger characterized by energy-efficient foraging flight, exploits large areas in search of unpredictable carcasses^34^,^35^,^36^,^37^,^38^. Individual breeding status and the temporally variable food requirements (more meat is required during the chick-rearing period^39^) could explain the differences in the use of space. Our findings show asymmetries in space use between territorial and non-territorial individuals. Breeding individuals exploit smaller areas (K90 63 ± 59.5 km^2^) in contrast to non-territorial individuals, which cover areas of 1818–11 616 km^2^ depending on age-class (Table 4). In a previous study^25^ based on nine pre-adult individuals in the Pyrenees tracked with satellite telemetry (but not GPS technology), the MCP obtained ranges of 945–19 691 km^2^. A similar pattern^26^ was observed in the subspecies *Gypaetus barbatus meridionalis* in South Africa in which breeding individuals covered on average 95 ± 19 km^2^, whilst non-territorial individuals covered areas of 10 540–25 985 km^2^. The differences are also significant in MCP, with 940.8 ± 1524.4 km^2^ for breeding individuals and 1566–13 270 km^2^ for non-breeding individuals. The South African subspecies had significantly larger MCP home range estimates, with home ranges for breeding adults around 5220 km^2^ and for non-breeding individuals at 21 151–40 961 km^2^. This marked difference between subspecies may result from factors such as conspecific attraction, the abundance of food resources and the modification of habitat quality by SFS, all of which may influence the movement ecology of Pyrenean bearded vultures^17^. It is generally assumed that, for vertebrates, foraging range size is inversely related to resource abundance and spatio-temporal predictability^40^. In other large-bodied raptors, home ranges vary according to prey density and individual reproductive status, with habitat quality acting as the main regulatory mechanism of space use^41^,^42^. This is the case of the bearded vulture in the Pyrenees, an area characterized by abundant natural food resources (i.e. wild and domestic ungulates^32^,^33^) that also possesses a network of SFS providing additional food. These predictable sites attract concentrations of dozens of mainly pre-adult individuals^29^. These differences may have important conservation implications given the threatened status of the species in Europe and the need to connect subpopulations so as to reduce the extinction risk. In this sense, movements of the non-territorial population of Pyrenean bearded vultures differ from those in other reintroduced subpopulations (Andalusia and Alps), in which there are greater range movements^17^. Several non-mutually exclusive factors could explain the differences found between wild and reintroduced individuals^17^. Firstly, captivity (individuals from Alps and Andalusia) can relax selective pressures, change the direction of selection or impose completely novel pressures, thereby provoking noticeable behavioural changes. In addition, the sharp decline of the European bearded vulture population may have had serious genetic consequences and the genetic differences found in the Pyrenean population could be linked to individual dispersal propensity. Finally, human activities can alter selective environments and disassociate certain behavioural and life history decisions from the outcomes that are normally expected of them, thereby creating evolutionary traps and thus rapid environmental changes that result in shifted behavioural decisions^17^. The small home ranges of Pyrenean bearded vultures limit the geographical expansion of the species. For example, over the last 20 years, only a small expansion of the distribution area has occurred in the Pyrenees (40 km westwards)^31^ despite its population growth (from 53 territories in 1991 to 130 in 2015). Habitat quality and conspecific attraction regulated by SFS may be responsible for the shrinkage of territories, the density-dependent effects on breeding output and the lack of geographical expansion. Thus, actions designed to extend the predictable food sources provided by the network of SFS have been proposed as the main actions to be employed to facilitate the expansion process^17^,^31^.

Our results show sexual and age-class asymmetries in both the daily mean movements and maximum distance travelled. Non-territorial females travelled less far than males but territorial females moved to more distant places than males. In addition, territorial females exploited larger areas (MCP and K50) than males. The potential drivers of this pattern are difficult to understand from ecological and evolutionary points of view since the division of parental roles in breeding bearded vultures is equitable^43^ and so *a priori* we would expect similar foraging distances in the sexes in the search for food resources. The shorter distances travelled by non-territorial females could be explained by their greater dependence on predictable food sources or patches, which might reduce their movements. However, this pattern will also be applicable to breeding individuals visiting distant SFS, in which foraging movements are compensated for by the predictability (spatial and temporal) of the available food resource. In fact, females of Egyptian vultures (*Neophron percnopterus majorensis*) have been reported to visit supplementary feeding stations more frequently than males^44^. In other species such as the California Condor (*Gymnogyps californianus*) monthly home ranges of adults were significantly larger than those of immatures; however, males and females had monthly home ranges of similar sizes throughout the year and breeding adults did not differ from non-breeding adults in their average monthly home range size^45^. On the contrary, in Eurasian griffon vultures (*Gyps fulvus*) no differences were found in home ranges between adults and immatures^22^. This differentiated foraging behaviour in obligate avian scavengers suggests that selective foraging habitat varies between species and age classes. Although predictable resources, lack of experience and reproductive constraints could partially explain such differences, our findings show that non-territorial bearded vultures included more SFS in their home ranges than territorial individuals, although the intensity of use (frequency of nearby locations) of the SFS was similar. Inexperienced individuals included more SFS in their movements because they act as predictable food supplies and as a site for interaction between conspecifics. Territorial birds used these sites less often, probably because they are able to exploit unpredictable but higher-quality prey more efficiently. This pattern could explain the territory-related differences in the use of SFS by bearded vultures. We found no differences between the sexes in territorial individuals in any of the buffers considered. The only significant result was found in non-territorial birds, in which male locations appeared more frequently in the 5-km buffers. However, these results could also be influenced by other factors such as the home range sizes of each individual, habitat quality (density of wild and domestic ungulates as the main food resource) and the spatial configuration of the SFS network. Indeed, the SFS network is not arranged in a spatially random way but is located mainly in high-quality breeding areas or near occupied breeding territories. Thus, used as a simple measure, the distance of locations from the SFS does not seem to be an objective proxy for determining their use. This could be due to the fact that the territorial birds in our study did not include SFS within their core areas but, by contrast, did have a higher frequency of locations near the SFS. Thus, if we take into account the smaller home range sizes of territorial bearded vultures, the use of large buffers (i.e. 5 km) as a surrogate measure for SFS use would seem to be unsuitable^25^. As a result, new methods and measures for studying the use of SFS by bearded vultures and the effects they have on vulture movements still need to be explored further.

With respect to the overlap with protected areas, our data suggest that around 44% of the home range of Pyrenean non-territorial bearded vultures lies within the network of protected areas, a figure that increases to 54% (K90) and 66% (K50) in territorial individuals. This implies that bearded vultures spend a significant amount of time foraging outside protected areas, thereby increasing the inherent risks of non-natural mortality due to anthropogenic factors^46^. Biodiversity conservation strategies are increasingly focused on regions outside protected areas, where animals face numerous anthropogenic threats and have to coexist with human settlements, livestock and agriculture^47^. As a result, an increase in conservation actions outside protected areas is a priority in any attempt to reduce the risk of non-natural mortality^46^. Of non-natural mortality factors, the risk of ingesting poison bait is one of the most serious factors affecting bearded vultures^36^ and other avian scavengers^48^,^49^,^50^,^51^,^52^ around the world. Other factors such as wind farms, lead poisoning and collisions with power lines have also been identified as drivers of non-natural mortality^36^,^38^,^53^. In addition, the authorization of the veterinary product Diclofenac in Spain in recent years is considered to be a potential threat to scavenger species. Carrion controls are needed to detect residues of veterinary pharmaceuticals that are potentially toxic to scavengers^54^,^55^.

Future decisions regarding the implementation of specific protected areas and the management of SFS should take advantage of the emerging discipline of movement ecology and be based on the robustness of data obtained via telemetry. For example, in addition to breeding sites as surrogates of priority protected areas, the data herein provided will aid the identification of important feeding areas (at a spatial and temporal scale) and thus help the conservation of specific species. This information will also allow us to improve the design and spatial distribution of the SFS network by evaluating the natural biomass provided by the habitat in relation to birds’ energetic requirements. Managers and policy-makers should consider these tools when seeking to implement evidence-based conservation actions^56^.

## Material and Methods

### Ethics Statement

All the work was conducted in accordance with relevant national and international guidelines, and conforms to all legal requirements. Captures and blood sample collection were carried out in compliance with the Ethical Principles in Animal Research. Thus, protocols, amendments and other resources were conducted in accordance to the guidelines approved by the Catalan Autonomous Government (Generalitat de Catalunya) following the R.D.1201/2005 (10 October 2005, BOE 21 October 2005) of the Ministry of Presidency of Spain. All experimental protocols were approved by the Catalan Autonomous Government and MAGRAMA (References 15546 and 25.306).

### Study species

The bearded vulture is a long-lived, threatened species that nests on mountain cliffs in Africa, Europe and Asia^57^. Declines in bearded vulture populations have been documented throughout its range, with habitat loss, direct persecution (poisoning and hunting) and reduced food availability being the main aggravating factors^58^,^59^. In Europe the species is restricted to the Pyrenees (170 breeding pairs), Alps (31 pairs), Crete (6 pairs), Corsica (5 pairs) and Andalusia (1 pair) (A. Margalida, unpubl. data). The primary risks to this population are the illegal use of poisons for predator control, lead intoxication, collisions with energy infrastructures and food shortages^58^,^60^.

### Capture and tracking

A total of 19 bearded vultures were fitted with satellite transmitters in the period 2006–2013. Transmitters were fitted to birds captured using radio-controlled bow-nets at feeding stations (*n* = 16), to fledglings in the nest (*n* = 1) or to individuals released after recovery at official rescue centres (*n* = 2).

Birds were assigned to one of four age classes according to previous demographic studies^61^: juveniles (birds in their first year of life), immatures (2–3 years), sub-adults (4–5 years) and adults (≥6 years). Individuals were sexed by molecular analysis of blood samples. Their territoriality, breeding status and annual breeding success were assessed through field observations.

We used solar-powered 70 g (GPS/PTT) satellite transmitters (Microwave Telemetry, Inc. Columbia, MD) to track movements. We attached transmitters using a backpack harness made from 0.64 cm Teflon ribbon (Bally Ribbon Mills, Bally, PA). We programmed transmitters to collect GPS locations (manufacturer’s estimated error: 18 metres) each hour from 6:00 to 21:00 UTC. Units on two birds (Dulantz and Revilla) were programmed to transmit a fix every two hours.

### Movement modelling

To study temporal variation in bearded vulture movement ecology, we divided the year into two seasons according to the breeding cycle: the breeding period (Br) from 1 January to 31 July and the non-breeding period (Non-br) from 1 August to 31 December.

Different measures were calculated to investigate daily movement patterns. The daily movement (km) was calculated summing the straight-line distances between successive locations on the same day. The maximum distance travelled per day (km) was calculated as the maximum distance from the initial daily location to any location reached on the same day. The average hourly distance between locations (km) was calculated as the straight-line distance between two consecutive locations and standardized in relation to the hours spent between locations. We limited the hourly frequency between locations to a maximum of two hours (i.e. seven or more locations per day). The distance between locations was calculated using basic trigonometry.

We used dynamic kernel models in the adehabitatHR package to calculate utilization distributions (UD)^62^. This package was run in Rstudio 0.99 and R version 3.0.3^63^ in combination with ArcGIS 9.1 (ESRI 2003) to build UD surfaces. Annual and seasonal UDs were estimated per bird using the ad hoc method as a smoothing parameter for comparison with previous studies^26^. Resolution of UD surfaces was established at 1 ha. We calculated the 90% (K90) and 50% kernel (K50) density contours. K90 represents the home range and K50 the core area of activity. Birds with fewer than 50 locations per season or 15 per month were removed from the analyses. Additionally, we calculated the Minimum Convex Polygon (MCP), which is traditionally used as a measure of the maximum area of activity. All values are presented as mean ± standard deviation (sd).

### Overlap of foraging movements with protected areas

We calculated the core area (K50) and home range (K90) of non-territorial and territorial birds separately. Protected area cover for France, Andorra and Spain was obtained from government and institutional online sources (https://inpn.mnhn.fr, http://www.iea.ad and http://www.idee.es, respectively). For this study, we only took into account the following protected area categories: National and Natural/Regional Parks and Special Protected Area (SPAs, Birds Directive 2009/147/CE) due their importance for bird protection.

### Statistical performance

We analyzed age- and sex-related differences in distance travelled per day, maximum distance per day, mean hourly distance and home-range size estimators using a Wilcoxon rank sum test. We analyzed each relationship independently.

After assessing the combined effect, we evaluated the effect of four factors: age, sex, territorial status (territorial vs. non-territorial) and season (breeding season vs. non-breeding season) on home range size using generalized linear mixed models (GLMM)^64^. We built nested models that included each factor separately (i.e. age), factors combined with other factors (i.e. age + season), and the interactions between factors (i.e. age*season). We included a null model (1 + random factor) to evaluate the performance of the models. To avoid pseudoreplication, we included as a random factor individual identity and year. GLMMs were fitted by maximum likelihood (ML) using the function “glmer” in “lme4” R package^65^ with a Gaussian error distribution and log link. Akaike’s information criterion (AIC) was used to compare the nested models with null models; differences in goodness-of-fit between models were evaluated by a log-likelihood ratio test calculated using the R function anova^66^. Additionally, to detect differences in factor categories, pairwise comparisons between age classes and breeding season were performed using the “multcomp” package^67^. *P* values were adjusted using the Tukey method, the default for pairwise comparisons between adjusted means.

We used the paired Wilcoxon rank sum test to study differences in territorial status in the percentage of locations at 0.5 km, 1 km and 5 km from a SFS and the number of SFS within K20, K50 and K90. For these analyses, juvenile birds were excluded from the non-territorial class due to their strong dependence on the parental territory during the first months after fledgling. All statistical procedures were performed in R version 3.0.3^63^.

## Additional Information

**How to cite this article**: Margalida, A. *et al*. Spatial and temporal movements in Pyrenean bearded vultures (*Gypaetus barbatus*): Integrating movement ecology into conservation practice. *Sci. Rep.* **6**, 35746; doi: 10.1038/srep35746 (2016).

## Supplementary Material

Supplementary Information

## Figures and Tables

**Figure 5:**
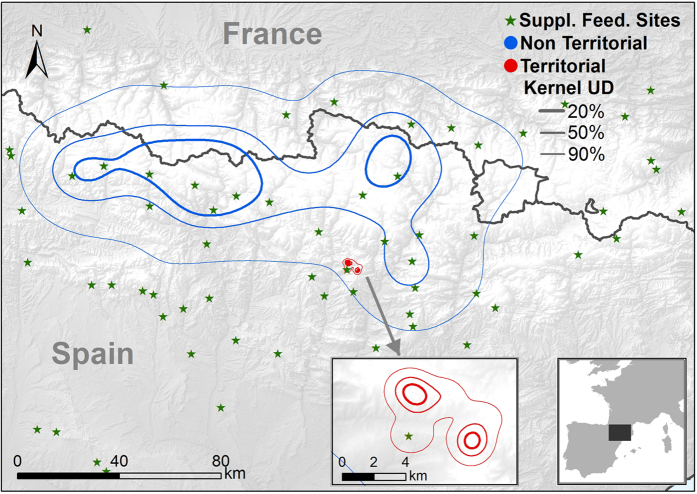
Example of bearded vulture *Gypaetus barbatus* ranging behaviour tracked in the Pyrenees in relation to location of Supplementary Feeding Sites (Suppl. Feed. Sites). We show utilization density areas UD kernel 90% (thick line), 50% (medium line) and 20% (thin line) for territorial bird (red) and non-territorial bird (blue) and Suppl. Feed. Sites (green stars). Maps were created in Rstudio 0.99 (https://www.rstudio.com) and R 3.0.3 (http://www.R-project.org) in combination with ArcGIS 9.1 (http://www.esri.com/ see Material and Methods).

**Table 1:** Detailed features of the 19 bearded vultures *Gypaetus barbatus* tracked by GPS/PTT between 2006 and 2014 in the Pyrenees.

Name	Sex	Age^1^	no. ID	Capture Date	End Date	Days	Locs	Territ.
Tossal	F	Ad	66996	29/11/2006	28/12/2006*	29	57	Yes
Gervàs	F	Ad	28666^1^	08/05/2007	31/03/2009^†^	693	2603	Yes
Morreres	M	Juv	78013	08/11/2007	11/09/2012*	1769	6132	No
Cabó	F	Ad	78017^1^	14/11/2007	26/07/2008^†^	255	658	Yes
Noah	F	Ad	78016	06/04/2008	17/09/2008*	165	382	No
Garrotxa	F	Sub	81093	06/05/2008	24/06/2013*	1875	5588	(2012)
Subfli	F	Sub	81092	06/05/2008	27/04/2012*	1453	4798	(2012)
Batín	M	Ad	81094	20/05/2008	31/12/2014	2184	7886	Yes
Sofia	F	Ad	78017^1^	18/11/2008	27/03/2012*	1225	2720	No
Andreia	F	Ad	86498^1^	03/03/2009	27/09/2009^†^	208	660	Yes
Min	F	Sub	28666^1^	14/05/2009	31/12/2014	1824	5858	(2014)
Adrian	M	Sub	86499	14/05/2009	30/12/2014	1824	7066	(2012)
Nicky	M	Sub	86500	03/06/2009	14/05/2014*	1806	3907	(2011)
Sasi	M	Juv	NA_55	01/08/2007	05/06/2008*	309	575	No
Jairo	M	Sub	86501	23/11/2010	31/12/2014	1266	5027	No
Isaac	M	Sub	78018	25/11/2010	20/01/2014*	1152	5350	No
Pocholo	M	Ad	86498^1^	25/07/2011	31/12/2014	1022	4911	No
Revilla	F	Sub	17756	23/04/2013	24/11/2013^†^	215	780	No
Dulantz	M	Ad	21377	25/04/2013	22/10/2014^†^	545	1716	No

We include name, sex (F female; M male), age when the bird was captured (Ad: adult; Sub: subadult; Imm: immature; Juv: juvenile), PTT identification number (no. Id), date of capture, date of the end of the monitoring, number of days of monitoring (no. days), number of locations (no. locs) and territoriality or year of territory settlement.

^1^PTT recovered and fitted to other new individual. ^†^Dead bird. *Transmitter failure.

**Table 2:** Mean distance travelled per day (“Mean daily travel.”), maximum distance reached per day (“Max dist reached”), average hourly distance between locations (“Avg. hourly dist.”) and number of tracked bearded vultures *Gypaetus barbatus* per age class (n) are displayed.

		n	Mean daily travel. (km)	Max dist. reached (km)	Avg hourly dist. (km)
Adult	Terr	8	23.8 ± 20.9 (1.6–161.3)	8.2 ± 12 (0.5–104.6)	2.5 ± 3.9 (0–63.2)
	No Terr	12	46.1 ± 41.4 (0–259.5)	27.3 ± 27.3 (0–159.8)	4.5 ± 7.3 (0–69.2)
Subadult		9	38.3 ± 39.4 (0.4–221.1)	25.9 ± 28.8 (0.1–176.2)	4.5 ± 7.3 (0–49.4)
Immature		2	31.2 ± 29.7 (2.8–141.1)	19.8 ± 23.5 (0.9–139)	3.7 ± 6.2 (0–36.4)
Juvenile		2	8.4 ± 7.3 (0.1–21.8)	5.7 ± 5.2 (0.2–17.2)	1.1 ± 2.1 (0–12.2)
Adult No Terr F		6	37.4 ± 34 (0–193.6)	24.3 ± 24.5 (0–142.2)	4.6 ± 7.6 (0–69.2)
Adult No Terr M		8	47.2 ± 44 (0–259.5)	27.9 ± 29 (0–159.8)	4.1 ± 6.6 (0–49.1)
Adult Terr F		5	26 ± 23.6 (1.6–151.5)	12 ± 18.2 (0.5–104.6)	3 ± 5 (0–63.2)
Adult Terr M		3	22.8 ± 19.6 (1.6–161.3)	6.6 ± 7.6 (0.6–99.5)	2.3 ± 3.3 (0–56.8)

Data of territorial and non-territorial adults are also represented by sex. All data show mean ± standard deviation and range (minimum –Maximum). Terr: territorial; No Terr: non-territorial; F: female; M: male.

**Table 3:** Annual home range size (km^2^) of bearded vultures *Gypaetus barbatus* tracked in the Pyrenees from 2006 to 2014 by age class and territorial status.

		n	MCP	Kernel 90	Kernel 50
Adult	Terr	8	940.8 ± 1524.4	63 ± 59.5	13.8 ± 13.8
	No Terr	12	13 270.3 ± 7743.7	11 406.4 ± 5441.5	3010.6 ± 1749.6
Subadult		9	11 629.9 ± 9298.2	11 616.8 ± 7849.6	3696.6 ± 2814.9
Immature		2	11 954.8 ± 9852.8	9295.3 ± 6880.1	2616.5 ± 2004.4
Juvenile		2	1566.4 ± 978.2	1818.2 ± 1758.9	488.3 ± 591.3
Adult No Terr F		6	13 785.5 ± 9958.7	11 604.3 ± 6673.8	3160.2 ± 2187.2
Adult No Terr M		8	10 504.3 ± 5067.6	10 175.4 ± 5815.2	2945.4 ± 2032.1
Adult Terr F		5	1049.7 ± 1819.1	46.3 ± 32.0	9.6 ± 8.0
Adult Terr M		3	723.1 ± 702.4	96.4 ± 87.4	22.3 ± 19.2

We show the minimum convex polygon (MCP), 90% kernel, 50% kernel and the number of birds per age class (n). All data show mean ± standard deviation. Data of territorial and non-territorial adults are also represented by sex. Terr: territorial; No Terr: non-territorial; F: female; M: male.

**Table 4:** Multiple comparisons of means: Tukey contrasts for GLMM of territorial status (No Terr = non-territorial; Terr = Territorial) of home range size (K90) and core area size (K50), and contrasts according to the age class of bearded vultures *Gypaetus barbatus* for each home range level (K90 and K50).

	Estimate	SE	Z	P
No Terr vs Terr				
K50	0.074	0.003	25.23	**<0.001**
K90	−0.014	0.160	−0.09	0.931
Age class at K50				
imm-ad	−0.294	0.424	−0.694	0.890
juv-ad	−1.861	0.601	−3.096	**0.009**
sub-ad	0.047	0.197	0.241	0.994
juv-imm	−1.567	0.573	−2.735	**0.027**
sub-imm	0.342	0.406	0.842	0.820
sub-juv	1.908	0.589	3.239	**0.005**
Age class at K90				
imm-ad	−0.303	0.003	−90.74	**<0.001**
juv-ad	−1.629	0.003	−487.36	**<0.001**
sub-ad	0.003	0.003	1.00	0.73
juv-imm	−1.325	0.005	−280.45	**<0.001**
sub-imm	0.306	0.005	64.87	**<0.001**
sub-juv	1.632	0.005	345.30	**<0.001**

Age class: juvenile (juv), immature (imm), subadult (sub) and adult (ad).

**Table 5:** Differences in the number of supplementary feeding sites (SFS) located within the UD kernel (K20, K50 and K90) and percentage of locations at 0.5 km, 1 km and 5 km from bearded vulture *Gypaetus barbatus* SFS.

	SFS inside			Percent of locations (%)		
	K20	K50	K90	0.5 km	1 km	5 km
No Terr	2.76 ± 2.33	7.62 ± 5.44	22.58 ± 10.63	1.96 ± 1.95	3.61 ± 3.20	29.99 ± 17.03
Terr	0 ± 0	0.14 ± 0.56	0.71 ± 0.56	2.53 ± 2.82	6.24 ± 5.60	67.56 ± 36.78

Data for territorial (Terr) and non-territorial (No Terr) birds are shown. For all data, mean ± standard deviations are given.
