# Supplementary material for: Spatial and temporal movements in Pyrenean bearded vultures (Gypaetus barbatus): Integrating movement ecology into conservation practice
Source: Sci Rep. 2016 Oct 25;6:35746. doi: 10.1038/srep35746 (PMC5078842; doi:10.1038/srep35746)
Supplement: Supplementary Information [file srep35746-s1.pdf]

Supplementary Information

Spatial and temporal movements in Pyrenean bearded vultures (*Gypaetus barbatus*): Integrating movement ecology into conservation practice

Antoni Margalida*[1,2], Juan Manuel Pérez-García[1,3], Ivan Afonso[4], Rubén Moreno-Opo[5]

[1] *Department of Animal Science, Faculty of Life Sciences and Engineering, University of Lleida, 25198 Lleida, Spain. Tel: (+34) 973 003 721. Email: amargalida@prodan.udl.cat*

[2] *Division of Conservation Biology. Institute of Ecology and Evolution. University of Bern. CH-3012. Bern, Switzerland*

[3] *Department of Applied Biology, University Miguel Hernández, E-03202 Elche, Spain.Tel: (+34) 965 222 123 Email: jperez@umh.es*

[4] *Conselh Generau d'Aran, Passeg dera Libertat, 16 E-25530 Vielha, Spain. Tel: (+34) 973 641 801. Email: ivan.afonso@tetrao.org*

[5] *Evolution and Conservation Biology Research Group. University Complutense of Madrid. E-28049 Madrid, Spain. Email: rmorenoopo@gmail.com*

Table S1. Mean daily movement (km), maximum distance moved per day (km) and average hourly distance between fixes (km) of GPS-tracked bearded vultures *Gypaetus barbatus* in the Pyrenees from 2006 to 2014. Differences between breeding season and non-breeding season and number of tracked bearded vultures per age class (n) are displayed. Data on territorial and non-territorial adults are also represented by sex. All data are shown as mean ± standard deviation and range value. Terr: territorial; No Terr: non-territorial; F: female; M: male.

		Breeding				Non-breeding			
		**n**	**Mean daily mov**	**Max distance day**	**Average hourly**	**n**	**Mean daily mov**	**Max distance day**	**Average hourly**
**Adult**	**Terr**	6	3.6 ± 8.4 (0 - 104.6)	17 ± 23.2 (0 - 121.7)	2.5 ± 3.7 (0 - 46.5)	8	3 ± 7.3 (0 - 99.6)	16 ± 14.3 (0,0 -0)	2.4 ± 4 (0,0 -63.2)
	**No Terr**	12	13.8 ± 21.6 (0 - 159.8)	58.6 ± 42.7 (0.1 - 270.3)	4.6 ± 7.5 (0 - 69.2)	12	11.3 ± 16.7 (0 - 157.6)	61.9 ± 42.7 (0,0 -0.3)	4.3 ± 7 (0,0 -54.7)
**Subadult**		7	14.6 ± 23.1 (0 - 176.2)	66.5 ± 59.7 (0 - 269.3)	4.7 ± 7.7 (0 - 48.2)	9	12.5 ± 18.7 (0 - 130.2)	57.6 ± 39.3 (0,0 -0.1)	4.3 ± 6.8 (0,0 -49.4)
**Immature**		2	8.3 ± 13.3 (0 - 73.3)	81.9 ± 58.2 (0.1 - 197.3)	3.2 ± 5.4 (0 - 33.4)	1	11.4 ± 20.1 (0 - 139)	62 ± 50 (0,0 -0.2)	4.2 ± 6.9 (0,0 -36.4)
**Juvenile**		0	-	-	-	2	3 ± 4.3 (0 - 17.2)	51.9 ± 13.3 (0,0 -6.5)	1.1 ± 2.1 (0,0 -12.2)
**Adult No Terr F**		5	12.5 ± 18.4 (0 - 141.1)	56.2 ± 37.6 (0.2 - 163.7)	4.4 ± 6.8 (0 - 49.1)	5	10.5 ± 15.2 (0 - 117.8)	64.4 ± 38.3 (0,0 -0.3)	3.6 ± 6.2 (0,0 -48.9)
**Adult No Terr M**		7	14.7 ± 23.3 (0 - 159.8)	60.1 ± 45.6 (0.1 - 270.3)	4.7 ± 7.9 (0 - 69.2)	7	11.7 ± 17.3 (0 - 157.6)	60.8 ± 44.4 (0,0 -0.5)	4.6 ± 7.3 (0,0 -54.7)
**Adult Terr F**		3	6.3 ± 14.2 (0 - 104.6)	30.3 ± 34.1 (0.1 - 121.7)	3.2 ± 5 (0 - 46.5)	5	4.1 ± 10.2 (0 - 99.6)	26.1 ± 11.8 (0,0 -0.5)	2.5 ± 5 (0,0 -63.2)
**Adult Terr M**		3	2.4 ± 2.9 (0 - 46)	11.1 ± 12.1 (0 - 47.8)	2.2 ± 3 (0 - 35.8)	3	2.7 ± 6.1 (0 - 99.5)	13.1 ± 13.7 (0,0 -0)	2.4 ± 3.7 (0,0 -56.8)

Table S2. Home range size (km^2) of GPS-tracked bearded vultures *Gypaetus barbatus* in Pyrenees from 2006 to 2014. Results were sorted by season, age and territorial status (km^2). Data on territorial and non-territorial adults are also represented by sex (female F and male M). We show minimum convex polygon (MCP), UD kernel 90%, UD kernel 50% and number of birds/season per age class (n).

		Breeding				Non-breeding			
		n	MCP	Kernel 90	Kernel 50	n	MCP	Kernel 90	Kernel 50
**Adult**	**Terr**	19	670.1 ± 1199.5	53.4 ± 46.3	12.5 ± 13.3	20	438.2 ± 1,146.9	63.8 ± 68.7	14.8 ± 15.6
	**No Terr**	34	10762.6 ± 6573.7	10346.1 ± 5861.1	2710.9 ± 1869.4	29	10,692.1 ± 5,141.6	1,1240.6 ± 6,389.1	3,119.9 ± 2,145.1
**Subadult**		11	10375.2 ± 9126.7	11981.5 ± 9048.4	3722.4 ± 3053.9	13	8,577.7 ± 6722.9	10626.2 ± 8120.5	3,405.4 ± 2,790.5
**Immature**		3	8984.5 ± 7540.2	9917.9 ± 7818	2803.6 ± 2297.2	2	13,131.4 ± 1512.6	13584 ± 350.8	4,299.8 ± 3.2
**Juvenile**		0	-	-	-	2	1,566.4 ± 978.2	1819.3 ± 1759.3	489.6 ± 592.4
**Adult No Terr F**		17	12690.4 ± 7654.2	11955.1 ± 6298.9	3055.2 ± 2063.9	16	12608.3 ± 5366.3	5326.8 ± 2835.4	2835.4 ± 1708.7
**Adult No Terr M**		17	8834.7 ± 4758.4	8636.5 ± 4991.4	2345 ± 1623.2	13	8481.1 ± 3999.9	7663.4 ± 3448.1	3448.1 ± 2594.1
**Adult Terr F**		14	599.2 ± 1332.3	50.2 ± 51	11.2 ± 14.7	14	550.6 ± 1366.9	52.7 ± 52.9	12.3 ± 12.6
**Adult Terr M**		5	868.5 ± 799.5	62.4 ± 32.6	16 ± 8.4	6	176.1 ± 151.1	89.6 ± 97.7	20.5 ± 21.4

Table S3. Summary of generalized linear mixed-effect model GLMM of the home range size in Pyrenean bearded vulture *Gypaetus barbatus* against biological variables and their interactions correlates. We contrasted GLMM models for two home range size estimators (K50 and K90) in a) all birds, b) territorial birds and c) non-territorial birds by log-likelihood test. All GLMMs included tracked bird "Indiv." and year "Year" as random effect. Interaction models of "Age" with "Sex" and "Age" with "Season" did not converge with any home range estimator (K90 and K50). Bold type denotes significant results. We show the combination of parameters for each model, degrees of freedom Df, Akaike's Information Criterion AIC, log-likelihood logLik, Chi square (χ^2) and p-value (*P*).

**a) All birds**	**Df**	**AIC**	**logLik**	χ^2	***P***
K50 ~ 1 + (1 | Indiv.) + (1 | Year)	4	2021.5	-1006.7		
K50 ~ Sex + (1 | Indiv.) + (1 | Year)	5	2023.3	-1006.6	0.19	0.665
K50 ~ Season + (1 | Indiv.) + (1 | Year)	5	2023.4	-1006.7	0	1
**K50 ~ Terr + (1 | Indiv.) + (1 | Year)**	**5**	**1940.8**	**-965.42**	**82.56**	**<0.001**
K50 ~ Season + Sex + (1 | Indiv.) + (1 | Year)	6	2025.2	-1006.6	0	1
K50 ~ Season * Sex + (1 | Indiv.) + (1 | Year)	7	2026.7	-1006.4	0.51	0.476
**K50 ~ Age+ (1 | Indiv.) + (1 | Year)**	**7**	**2020.5**	**-1003.3**	**6.23**	**<0.001**
**K50 ~ Sex * Terr + (1 | Indiv.) + (1 | Year)**	**7**	**1944.4**	**-965.22**	**76.06**	**<0.001**
K50 ~ Season * Terr + (1 | Indivi.) + (1 | Year)	7	1944.7	-965.36	0	1
K50 ~ Age+ Sex + Season + (1 | Indiv,) + (1 | Year)	9	2024.2	-1003.1	0	1
**K50 ~ Age+ Sex + Season + Terr + (1 | Indiv.) + (1 | Year)**	**10**	**1942.6**	**-961.28**	**83.66**	**<0.001**
K90 ~ 1 + (1 | Indiv.) + (1 | Year)	4	2362.8	-1177.4		
K90 ~ Sex + (1 | Indiv.) + (1 | Year)	5	2364.5	-1177.2	0.34	0.557
**K90 ~ Season + (1 | Indiv.) + (1 | Year)**	**5**	**2364.4**	**-1177.2**	**0.07**	**<0.001**
**K90 ~ Terr + (1 | Indiv.) + (1 | Year)**	**5**	**2280.6**	**-1135.3**	**83.71**	**<0.001**
K90 ~ Season + Sex + (1 | Indiv.) + (1 | Year)	6	2366.1	-1177	0	1
K90 ~ Season * Sex + (1 | Indiv.) + (1 | Year)	7	2367.8	-1176.9	0.21	0.643
**K90 ~ Age + (1 | Indiv.) + (1 | Year)**	**7**	**2363**	**-1174.5**	**4.81**	**<0.001**
**K90 ~ Sex * Terr + (1 | Indiv.) + (1 | Year)**	**7**	**2284.6**	**-1135.3**	**78.5**	**<0.001**
**K90 ~ Season * Terr + (1 | Indiv.) + (1 | Year)**	**7**	**2284.5**	**-1135.3**	**0.01**	**<0.001**
K90 ~ Age + Sex + Season + (1 | Indiv.) + (1 | Year)	9	2366.4	-1174.2	0	1
**K90 ~ Age + Sex + Season + Terr + (1 | Indiv.) + (1 | Year)**	**10**	**2282.8**	**-1131.4**	**85.5**	**<0.001**

b) Territorial birds

	Df	AIC	logLik	χ^2	*P*
K50 ~ 1 + (1 | Indiv.) + (1 | Year)	4	258.5	-125.25		
K50 ~ Sex + (1 | Indiv.) + (1 | Year)	5	260.41	-125.2	0.09	0.759
**K50 ~ Season + (1 | Indiv.) + (1 | Year)**	**5**	**260.32**	**-125.16**	**0.09**	**<0.001**
K50 ~ Season + Sex + (1 | Indiv.) + (1 | Year)	6	262.24	-125.12	0.08	0.782
K50 ~ Season * Sex + (1 | Indiv.) + (1 | Year)	7	263.97	-124.98	0.27	0.602
K90 ~ 1 + (1 | Indiv.) + (1 | Year)	4	376.23	-184.12		
K90 ~ Sex + (1 | Indiv.) + (1 | Year)	5	378.05	-184.02	0.19	0.665
K90 ~ Season + (1 | Indiv.) + (1 | Year)	5	378.22	-184.11	0	1
K90 ~ Season + Sex + (1 | Indiv.) + (1 | Year)	6	380.04	-184.02	0.19	0.665
K90 ~ Season * Sex + (1 | Indiv.) + (1 | Year)	7	382.04	-184.02	0.00	0.989

c) Non-territorial birds

	Df	AIC	logLik	χ^2	*P*
K50 ~ 1 + (1 | Indiv.) + (1 | Year)	4	1640	-816		
K50 ~ Sex + (1 | Indiv.) + (1 | Year)	5	1641.3	-815.63	0.74	0.388
K50 ~ Season + (1 | Indiv.) + (1 | Year)	5	1641.9	-815.95	0	1
K50 ~ Season + Sex + (1 | Indiv.) + (1 | Year)	6	1643.2	-815.58	0.75	0.388
K50 ~ Season * Sex + (1 | Indiv.) + (1 | Year)	7	1643.9	-814.95	1.26	0.261
**K50 ~ Age + (1 | Indiv.) + (1 | Year)**	**7**	**1638.4**	**-812.21**	**5.49**	**<0.001**
K50 ~ Age + Sex + Season + (1 | Indiv.) + (1 | Year)	9	1641.4	-811.72	0.98	0.612
K90 ~ 1 + (1 | Indiv.) + (1 | Year)	4	1857.2	-924.58		
K90 ~ Sex + (1 | Indiv.) + (1 | Year)	5	1858.6	-924.28	0.59	0.442
K90 ~ Season + (1 | Indiv.) + (1 | Year)	5	1859.1	-924.57	0	1
K90 ~ Season + Sex + (1 | Indiv.) + (1 | Year)	6	1860.5	-924.27	0.59	0.442
K90 ~ Season * Sex + (1 | Indiv.) + (1 | Year)	7	1862	-924.02	0.50	0.481
**K90 ~ Age + (1 | Indiv.) + (1 | Year)**	**7**	**1856**	**-920.98**	**6.08**	**<0.001**
K90 ~ Age + Sex + Season + (1 | Indiv.) + (1 | Year)	9	1859.5	-920.76	0.44	0.802
